# Assessing Academic Library Perfor-mance: A Handbook

**DOI:** 10.5195/jmla.2022.1595

**Published:** 2022-07-01

**Authors:** Jenessa M. McElfresh

**Affiliations:** 1 jmcelfre@uthsc.edu, University of Tennessee Health Science Center, Memphis, TN.

In an increasingly data-driven world, assessment is necessary to ensure the growth and future success of academic libraries. In *Assessing Academic Library Performance: A Handbook*, editor Holt Zaugg presents a thorough overview of assessment theory, techniques, and case studies that can be utilized by academic libraries to build or enhance their programs of assessment. As stated in the preface, this book aims to act as a “springboard to adopt, adapt, or create assessments to describe a library's value and to plan for improvement,” and it largely succeeds in this ambitious mission.

*Assessing Academic Library Performance* is organized into four categories based upon the common focus areas of assessment in an academic library: services, resources, spaces, and personnel relationships. Every section of the book begins with an introduction by Zaugg to that focus area, presenting rationale and potential uses of common methods of assessment before going more in-depth into the assessment design. These introductory chapters build upon the principles laid out in the particularly strong first chapter of the book, “Assessment Framework,” as these overview chapters tie in foundational knowledge of assessment frameworks to more specific types of assessment, assessment tools, and additional assessment components like library personas, library impact maps, and data inventories.

A particular strength of the book is Zaugg's use of the essential tasks of assessment as an organization strategy in establishing the background of assessment in each section's focus area. This model is based upon the Lee Library Assessment Framework, which effectively demonstrates the interconnected nature of library assessment projects to the university aims and goals. Each introductory chapter for the section is thus organized by the specific assessment designs, data collection methods, data analysis methods, and dissemination techniques that pertain to that topic. The consistent use of the Lee Library Assessment Framework as an organizational tool provides structure and clarity to concepts that might become otherwise confusing.

Following Zaugg's overviews to the services, resources, spaces, and personnel relationships sections, the ensuing chapters contain case studies from academic libraries that have designed and executed assessment projects in those focus areas. Case studies range from large-scale investigations across entire library systems to smaller projects that target a unique unit or library. With some exceptions, most of the included case studies are from large public research universities, though the data collection methods can be scaled up or down in scope as needed. While some case studies contain images, graphics, data visualization, appendices, or suggested further readings, others are simpler and focus on the library's experiences with that assessment project. Case studies in the “Space Assessments” section are particularly strong, containing resources and experiences that present numerous creative ideas for utilizing space assessments in library planning and advocacy.

Missing from the book are discussions on building equity and inclusion into assessment planning and mitigating bias from the assessment process. More information could be included on the impact of different types of assessment and project design on historically marginalized communities, as they directly pertain to ensuring diversity in populations sampled and creating equitable library environments for all. Particularly in sections on data collection methods, additional guidance on recognizing sources of bias, facilitating a no-retaliation data collection process, and utilizing different assessment types for libraries enhancing their diversity, equity, and inclusion commitments would greatly enhance this text.

*Assessing Academic Library Performance* is a useful launching point for an academic library's assessment needs. For academic health sciences and medical libraries, the uses of this book are directly tied to the stated purpose for academic libraries, the content of which contains numerous concepts and examples to generate creative and useful assessment plans in university library systems. Though aimed at use in academic libraries, hospital librarians have much to gain from this book in the implications for assessment projects to advocate in healthcare organizations for the necessity of both hospital library spaces and the expertise of hospital librarians.

As a practitioner-focused handbook, *Assessing Academic Library Performance* presents foundational knowledge and real-world case studies in an organized, easy-to-understand approach and contains additional resources and references to specific sources for readers that wish to go more in-depth. The handbook contains a wealth of knowledge and experiences that can be utilized by novices or experts as they continue to design, collect, analyze, and disseminate rich assessment projects that attest to the value of libraries.

